# P298: Underreporting of needlestick and sharps injuries at one tertiary care hospital in Saudi Arabia

**DOI:** 10.1186/2047-2994-2-S1-P298

**Published:** 2013-06-20

**Authors:** VCD Syam, A Delos Santos, A Hakawi

**Affiliations:** 1 Infection Control, king Fahad Medical City, Riyadh, Saudi Arabia

## Introduction

Needlestick and sharp injuries (NSSI) represent a significant hazard for HCWs. The present study is the first to explore the magnitude and causes of underreporting of this type of occupational hazard at our institution.

## Objectives

To quantify the magnitude and causes of underreporting of needlestick and sharp injuries for the year 2011 and to identify and measure the gap between NSSI incidence rate reported through standard surveillance and that estimated from retrospective survey for the year 2011.

## Methods

Two methods of data collections were used; standard surveillance of NSSIs and a cross-sectional retrospective survey among Healthcare professionals.

The questionnaire was designed and validated by study team and consisted in a number of 19 questions. We used a non-probability quota sampling of HCP stratified proportionally to size by job category and departments .

Data entry and analysis were done in Microsoft Excel .Statistical analysis included frequency distribution, calculation of rates, Chi-square and Fisher test with significance level at 0.05.

## Results

There was a number of 73 NSSI reported through standard surveillance during 2011. The rate of NSSI was 1.60/100 HCPs. The self-administered questionnaire was distributed to a number of 1320 HCP. The response rate was 75%. A percent of 5.1% among respondents sustained NSSI during 2011. The identified gap between standard surveillance and cross sectional retrospective survey was 3.2 fold difference. The top circumstances in which injuries had occurred were: during needle disposal, during a medical procedure, during recapping the needle and, during surgery.

Only 72.5% of NSSIs had been reported. The reasons for not reporting the injury were: perception of low risk of infection, lack of time, fear of consequences, too much paper to fill or reporting purposes.

## Conclusion

The level of underreporting was around 27%.The causes of underreporting were perception of low risk of infection, lack of time and fear of consequences. The incidence rate of NSSI estimated by the cross sectional retrospective survey was 3.2 fold higher compared to that identified by standard surveillance.

## Disclosure of interest

None declared

